# Beyond metabolism: exploring the regulatory and therapeutic implications of lactate and lactylation in cancer-regulated cell death

**DOI:** 10.1038/s41419-026-08410-z

**Published:** 2026-01-22

**Authors:** Cong Chen, An Lin, Jiacheng Zhao, Xia Lin, Qianwei Ye, Jufeng Guo, Jian Liu, Aizhai Xiang

**Affiliations:** 1 Department of Breast Surgery, Affiliated Hangzhou First People’s Hospital, School of Medicine, Westlake University, Hangzhou, China; 2 Department of General Surgery, The Second People’s Hospital of Tonglu, Hangzhou, China

**Keywords:** Cell death, Post-translational modifications

## Abstract

Lactate, a key byproduct of glycolysis in tumor cells, has emerged as more than just a metabolic waste product. Increasing evidence reveals that lactate and its associated post-translational modification (PTM), lactylation, play multifaceted roles in regulating various forms of regulated cell death (RCD), thereby contributing to cancer proliferation, therapy resistance, and immune exclusion. Notably, evasion of RCD is a hallmark of cancer and targeting RCD may represent a promising therapeutic strategy for cancer treatment. In this review, we focus on summarizing the dual and context-dependent roles of both lactate and lactylation in modulating distinct types of RCD, including apoptosis, autophagy, ferroptosis, pyroptosis, and cuproptosis. Moreover, we further discuss how RCD processes impact lactate metabolism and highlight the therapeutic potential and current challenges of targeting the lactate-lactylation-RCD axis in cancer treatment.

## Facts

- Lactate, which is traditionally considered a metabolic waste product, plays an active regulatory role in modulating various forms of regulated cell death (RCD) and is thus involved in cancer progression.
- Lactylation, a recently discovered post-translational modification (PTM) derived from lactate, has been shown to affect gene expression and protein function involved in RCD pathways.
- Both lactate and lactylation exert dual and context-dependent effects on cell death and are capable of either promoting or inhibiting RCD, depending on the tumor type, cell subtype, metabolic status, and microenvironmental factors.
- Tumor cells can reshape lactate metabolism by modulating RCD programs, indicating a crosstalk between lactate metabolism and RCD.
- Targeting the lactate–lactylation–RCD axis may provide novel therapeutic opportunities for cancer treatment.

## Open Questions

- What are the determinants of the dual effects of lactate and lactylation on different forms of RCD, and how can these context-specific outcomes be reliably predicted or manipulated?
- What are the specific enzymes involved in lactylation, and how do they differ from other lysine modifications, such as acetylation?
- Can we develop strategies for dynamically and context-dependently modulating lactate metabolism in cancer treatment rather than simply suppressing its production or enhancing its clearance?

## Introduction

Lactate, which is traditionally regarded as a predominant byproduct of glycolysis, has long been misinterpreted as a metabolic waste [1]. However, accumulating evidence has revealed its key role as a metabolic and signaling molecule in tumor biology [2]. In tumor cells, glucose is metabolized predominantly into lactate even in the presence of sufficient oxygen, a metabolic hallmark known as aerobic glycolysis or the Warburg effect [3]. This reprogrammed metabolic phenotype enables cancer cells to utilize lactate as an alternative energy substrate to rapidly generate ATP and sustain their survival demands [4]. In addition, lactate modulates diverse signaling pathways within tumor cells and their microenvironments, influencing cancer cell progression and contributing to tumor microenvironment (TME) remodeling, thereby representing a promising target for therapeutic intervention in cancer treatment [5, 6].

Remarkably, a notable advancement in lactate research is the recent identification of protein lactylation, a post-translational modification (PTM) first observed on histones [7]. Subsequent studies revealed that this lactate-derived modification is not restricted to histones but also occurs on non-histone substrates, making it an attractive topic for cancer research [8]. By adding the lactyl group to specific lysine residues on proteins, lactylation alters protein expression, function, stability, and interactions, thereby serving as a significant PTM that regulates diverse cellular pathways and contributes to tumor initiation, progression, and metabolic adaptation [9].

Regulated cell death (RCD), also known as programmed cell death, is a genetically controlled and highly orchestrated process that plays fundamental roles in cell development and tissue homeostasis [10]. Dysregulation of RCD is closely linked to various pathological conditions, particularly cancer, as evasion of RCD is a hallmark of cancer that promotes tumor progression and treatment resistance [11]. As the field has advanced, several forms of novel RCD have been extensively explored and may represent promising therapeutic strategies for cancer treatment [12–16].

In the context of cancer, recent evidence indicates that both lactate and its associated PTM, lactylation, play crucial roles in modulating various forms of RCD, which has significant implications for cancer proliferation, therapeutic resistance, and immune exclusion. In this review, we summarize recent findings on how lactate and lactylation modifications affect distinct types of RCD, including apoptosis, autophagy, ferroptosis, pyroptosis, and cuproptosis, as well as the impact of RCD on lactate metabolism, highlighting a bridge between metabolic reprogramming and cell death pathways. Collectively, these insights offer promising avenues for the development of novel therapeutic strategies for cancer treatment in the future.

## Lactate production and transport

Cancer cells exhibit a unique metabolic adaptation known as the Warburg effect, where they preferentially rely on glycolysis for energy production despite the presence of oxygen [17]. Under aerobic conditions, pyruvate in normal cells is transported into the mitochondria and subsequently converted by the pyruvate dehydrogenase complex into acetyl-CoA, a key intermediate for the tricarboxylic acid (TCA) cycle and ATP synthesis [18, 19]. However, under hypoxic conditions or when glycolysis is upregulated (e.g., the Warburg effect in cancer cells), pyruvate is reduced to lactate via the activity of lactate dehydrogenase (LDH) in the cytoplasm [20, 21]. Besides, glutamine catabolism serves as an additional source of lactate production in cancer cells. Glutamine is first converted to glutamate in the cytoplasm, which is then metabolized into α-ketoglutarate (α-KG) and enters the TCA cycle. Within the TCA cycle, malate is a downstream metabolite of α-KG, which is released from the mitochondria and converted into pyruvate by malic enzyme (ME1) in the cytoplasm, ultimately leading to lactate production [2, 20].

Owing to their elevated metabolic activity, cancer cells generate significant levels of lactate in the cytosol, which requires efficient lactate export systems to prevent intracellular acidification and maintain sustained glycolytic flux [22]. Monocarboxylate transporters, primarily MCT1 and MCT4, were reported to mediate bidirectional lactate transport across the plasma membrane [23]. Under hypoxic conditions or in glycolytic tumor cells, the presence of low-affinity MCT4 facilitates lactate efflux to prevent intracellular acidification [24]. However, in oxidative tumor cells, high-affinity MCT1 mediates lactate uptake, enabling its utilization as a metabolic substrate for energy production [24]. Interestingly, this cooperative interaction, known as “metabolic symbiosis”, promotes lactate exchange between hypoxic and aerobic tumor cells, favoring tumor survival and metabolic flexibility in harsh microenvironments [20]. Indeed, the lactate-driven metabolic interaction between cancer cells and cancer-associated fibroblasts (CAFs) serves as another example [25]. Several studies have reported that MCT4 is upregulated in CAFs [26], leading to a significant increase in lactate secretion and providing metabolic support for oxidative cancer cells [27].

## Mechanisms of lactylation in brief

Lactylation, a newly identified PTM, has attracted significant interest in cancer research since its first discovery in 2019 [7]. The TME is enriched with lactate, which serves as a critical substrate for lactylation modifications in cancer cells. Like acetylation, L-lactate can be converted to L-lactyl-CoA through intracellular metabolic pathways. This L-lactyl-CoA then transfers its lactyl group to lysine residues on target proteins by specialized “writer” enzymes [28]. However, additional evidence shows that lactate modification can also be achieved through non-enzymatic reactions. Methylglyoxal, a byproduct of glycolysis, can form lactoyl-glutathione under the catalysis of glyoxalase I, which directly mediates the non-enzymatic lactylation modification process [29, 30]. Nevertheless, it has been shown that D-lactate is primarily responsible for this non-enzymatic process [28, 31].

It is currently believed that L-lactate is the most common type of lactate [32]. Therefore, in this review, all lactate and its associated lactylation modification refer to L-lactate unless otherwise noted. Recent studies have identified a variety of “writer” enzymes responsible for lactylation. Currently, GCN5 (KAT2A), P300 (KAT3B) and its homolog protein CBP (KAT3A), along with TIP60 (KAT5), HBO1 (KAT7), MOF (KAT8), AARS1, and AARS2, have been recognized as key lactylation “writer” proteins [28, 31]. Additionally, “eraser” enzymes, including histone deacetylases (HDAC1–3, HDAC8) and sirtuins (SIRT1–3) [28, 31, 33–35], play crucial roles in removing lactyl groups from modified proteins. Therefore, the “writer” and “eraser” enzymes function as a pair with opposing enzymatic activities, dynamically modulating lactylation levels in cells [36].

**Fig. 1:**
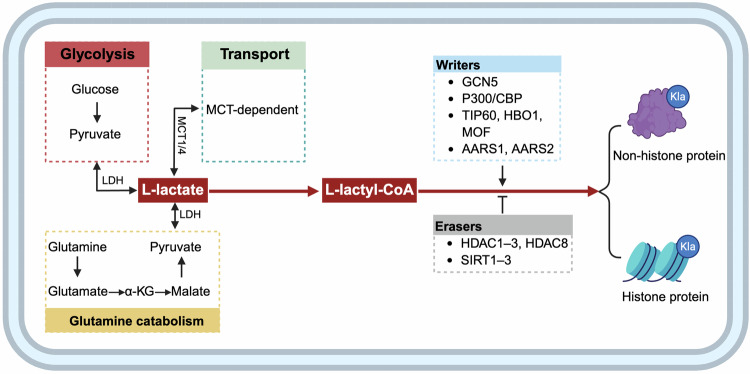
Core mechanisms of lactate production, lactate transport, and lactylation modification in cancer cells. Aerobic glycolysis is a metabolic hallmark of cancer cells and results in increased lactate production via LDH-mediated reduction of pyruvate. Glutamine metabolism also contributes to lactate synthesis through its conversion to α-ketoglutarate and downstream intermediates. Monocarboxylate transporters, particularly MCT1 and MCT4, regulate lactate exchange across the plasma membrane, supporting metabolic crosstalk in the TME. Intracellular lactate can be enzymatically converted into lactyl-CoA, which donates lactyl groups to lysine residues on histone and non-histone proteins. This post-translational modification is dynamically regulated by “writer” enzymes and “eraser” enzymes in tumor cells.

The study of lysine lactylation (Kla) was initially reported in histones [7], where it serves as a critical epigenetic modification that remodels chromatin architecture and regulates gene transcription in cancer cells [37, 38]. To date, numerous lactate modification sites with critical roles in tumor biology have been identified, such as H3K9, H3K14, H3K18, H3K56, H4K5, and H4K8 [5, 38]. Notably, subsequent studies confirmed that Kla also occurs in non-histone proteins, where it influences protein structure and function, thereby playing diverse regulatory roles in tumorigenesis [8]. The main processes of lactate production, transport and lactylation are summarized and presented in Fig. 1.

## Lactate, lactylation and apoptosis

Apoptosis is the most intensively studied form of RCD and serves as an important defense mechanism in preventing tumor development [39]. Recent preclinical and clinical studies have shown that activating apoptotic pathways or inhibiting anti-apoptotic signals in tumor cells with specific drugs can effectively induce apoptosis and exert anti-tumor effects [12, 40].

Specifically, recent studies have shown that lactate can also function as a regulatory molecule by modulating apoptosis in tumor cells through diverse signaling pathways, thereby influencing tumor progression. In non-small cell lung cancer (NSCLC), lactate treatment has been shown to inhibit FOXO3 expression through YTHDF2-mediated m6A modification [41]. This mechanism leads to an increase in the BCL-2 level and a decrease in the BAX level, thereby inhibiting the apoptosis program and enhancing cisplatin resistance both in vitro and in vivo [41]. Similarly, another study revealed that increased lactate could further induce the upregulation of SNAIL and TAZ, which interact with the AP-1 transcription factor to activate MRP1 expression, ultimately contributing to the inhibition of etoposide-induced apoptosis in NSCLC cells [42]. Consistent with these findings, lactate treatment attenuates erlotinib-induced apoptosis in NSCLC by triggering the downstream AKT pathway via its receptor protein GPR81 [43]. More specifically, under glucose-deprived conditions, lactate supplementation can inhibit tumor cell apoptosis in a dose-dependent manner [44, 45]. Notably, this effect was not related to the role of lactate in energy metabolism, but was achieved by activating the PI3K/AKT/mTOR pathway, leading to the upregulation of the anti-apoptotic protein BCL-2 [44, 45]. Taken together, the above findings suggest that lactate functions as a signaling molecule that promotes cancer proliferation and therapeutic resistance by inhibiting apoptosis.

However, several additional studies reported that lactate functions as an energy substrate, providing a necessary fuel source to help cancer cells evade apoptosis and survive under metabolic stress conditions. For example, under lactic acidosis conditions, research has revealed that lactate induces localized metabolic reprogramming that facilitates a switch from glycolysis to oxidative phosphorylation (OXPHOS) [46, 47]. This metabolic shift sustains high intracellular ATP levels, thereby enabling the tumor cells to resist glucose deprivation-induced apoptosis [46]. Similarly, Barnes et al. verified that treatment with the pan-AKT inhibitor uprosertib induced colon cancer cell apoptosis, which was significantly attenuated in the presence of lactate [48]. Mechanistically, elevated lactate concentrations in the medium enhanced OXPHOS in colon cancer cells, thereby compensating for the energy deficit resulting from uprosertib-induced glycolysis inhibition [48]. Therefore, lactate-mediated enhancement of OXPHOS can counteract the effects of glycolysis inhibition by anticancer therapies such as AKT inhibitors, ultimately reducing apoptosis and contributing to therapeutic resistance.

Notably, excessive intracellular lactate accumulation can also lead to detrimental effects. For example, Huang et al. demonstrated that high concentrations of lactate (such as 80 mM) can cause immediate death of A549 cells [44]. In addition, recent investigations have reported that certain small-molecule drugs can impair lactate export by inhibiting MCT4, resulting in excessive intracellular lactate accumulation [49, 50]. This accumulation lowers the intracellular pH, triggers calcium overload, and disrupts mitochondrial function, ultimately leading to tumor cell apoptosis [49, 50]. Strikingly, another study revealed that lactate accumulation triggered apoptosis in HeLa cells by activating P38 phosphorylation and upregulating the expression of apoptosis-related genes, whereas these effects were not detected in HEK293 and HCT116 cells [51]. Therefore, the pro-apoptotic effects of lactate are cell type-specific and may depend on distinct signaling pathways or metabolic contexts in different cell types.

Collectively, these findings highlight the bidirectional regulatory role of lactate in apoptosis, which largely depends on its source, concentration, and cellular context. A key determinant of lactate’s effect on cell apoptosis fate is whether it originates from exogenous supplementation or endogenous production during tumor adaptation to stress or therapy. When lactate is derived from tumor metabolic reprogramming, such as during chemotherapy or glucose-deprived conditions, it often serves as a cytoprotective factor. In this setting, lactate acts as a signaling molecule that suppresses apoptosis by activating pro-survival pathways such as PI3K/AKT/mTOR or GPR81-mediated signaling, or by functioning as an alternative energy substrate that sustains OXPHOS and maintains ATP levels under adverse environmental stress. In contrast, when lactate efflux is impaired, such as by pharmacological inhibition of MCT4, excessive intracellular lactate accumulation can lead to cytotoxic consequences. High lactate levels acidify the cytoplasm, induce calcium overload, and disrupt mitochondrial integrity, ultimately activating apoptotic cascades. Moreover, when lactate supplementation exceeds the buffering capacity of the cell, it can also trigger apoptosis.

Beyond the influence of lactate concentration and origin, the tumor genetic background introduces an additional layer of complexity to lactate-mediated apoptotic regulation. For instance, lactate significantly reduced etoposide-induced apoptosis in A549 but not in H1299 cells, a discrepancy explained by the fact that H1299 cells are p53-deficient. Importantly, restoration of p53 expression in H1299 cells reinstated the protective effect of lactate against etoposide-induced apoptosis [42]. Similarly, under glucose-deprived conditions, lactate supplementation markedly inhibited apoptosis in DU145, A549, and H1299 cells, but showed little effect in PC3 and U87-MG cells. Mechanistic analysis revealed that the two lactate-insensitive cell lines, PC3 and U87-MG, had lost PTEN activity [44], suggesting that the presence or absence of key tumor suppressors critically shapes cellular responsiveness to lactate-mediated survival signaling.

As reported, accumulating evidence indicates that lactylation also plays a crucial role in tumor progression by regulating apoptosis through different molecular mechanisms. For example, Li et al. demonstrated that lactylation of H4K12 suppressed SLFN5 promoter activity, which impedes triple-negative breast cancer (TNBC) cell apoptosis [52]. However, the mechanisms involved in this process need further elucidation. Furthermore, Wang et al. demonstrated that H3K18la promotes IDH3G expression via BZW2-dependent glycolysis, which is associated with apoptosis resistance in lung adenocarcinoma [53]. Specifically, H3K18la has also been shown to activate the PI3K/AKT signaling pathway, thereby inhibiting the apoptosis program and promoting the malignant biological behavior of tumor cells [54, 55]. Together, these studies suggest that lactylation not only rewires gene expression but also integrates into oncogenic signaling networks to sustain tumor cell survival.

Importantly, the impact of lactylation extends to therapeutic responses. Platinum-based chemotherapeutic agents, such as cisplatin, exert their effects primarily by inducing DNA damage and activating apoptosis. However, both histone and non-histone lactylation have been shown to enhance homologous recombination repair, ultimately diminishing drug-induced apoptosis [56, 57]. This reveals a direct mechanistic link between metabolic reprogramming and chemoresistance, suggesting that tumor cells may exploit lactylation as a defense strategy against DNA-damaging therapies. Moreover, Jin et al. applied a novel natural agent called honokiol to activate SIRT3, leading to the apoptosis of hepatocellular cancer (HCC) cells by removing the Kla modification from the CCNE2 protein [58]. This finding highlights the therapeutic potential of selectively targeting lactylation to overcome apoptosis resistance.

## Lactate, lactylation and autophagy

Autophagy is a dynamic and highly conserved process that maintains cellular homeostasis by degrading and recycling intracellular components through the lysosome-mediated pathway [59]. In the TME, tumor cells exploit autophagy as an adaptive mechanism in response to nutrient deprivation, hypoxia, and therapeutic interventions [13, 60]. Emerging evidence suggests that lactate, a critical metabolic byproduct in the TME, plays a pivotal role in modulating autophagy [61]. Herein, we aim to summarize the regulatory effects of lactate and lactate-driven lactylation on autophagic signaling in tumor cells, highlighting their implications for cancer progression and therapeutic resistance.

Consistent with the dual role of autophagy in cancer progression, lactate can either promote or inhibit autophagy. In HeLa cells and pancreatic cancer cells, lactate exposure has been shown to increase autophagic flux by promoting autophagosome formation [62]. Under glucose deprivation stress, elevated lactate levels promote the survival of 4T1 cells by inducing autophagy, which is associated with increased expression and enhanced stability of the P27 protein [45]. Nevertheless, an opposite opinion revealed that glucose depletion induces B16 melanoma cell death through autophagy in vitro, and further experiments confirmed that the acidic microenvironment induced by lactate can reverse this process by inhibiting autophagy, thereby promoting cancer cell survival [63]. These seemingly conflicting results may, in fact, represent different temporal phases of metabolic adaptation to glucose deprivation. In the B16 melanoma model, glucose deprivation triggers an acute metabolic crisis within the first 24 to 48 h, leading to excessive activation of autophagy that causes autophagic cell death [63]. Under this early-stage stress, lactate accumulation suppresses autophagy, likely through intracellular acidification that disrupts autophagosome–lysosome fusion, thereby preventing overactive autophagy and allowing cells to survive the acute energy shock [63]. By contrast, in the 4T1 breast cancer model, where glucose deprivation was prolonged up to 8 days, a biphasic pattern of autophagy regulation was observed under conditions of concurrent glucose deprivation with lactate exposure [45]. Within the first 48 h, LC3B-II expression decreased in the presence of lactate, indicating autophagy inhibition similar to the early-phase pattern observed in B16 melanoma cells [45]. However, after 48 h, LC3B-II expression markedly increased, reflecting a switch toward autophagy activation [45]. This late-phase induction of autophagy is consistent with a metabolic adaptation process, and lactate should be viewed not as a simple activator or inhibitor of autophagy but as a flexible rheostat that tumors exploit to optimize survival. When autophagy is protective, lactate tends to promote it; when autophagy threatens tumor viability, lactate suppresses it. In addition, another independent study showed that gefitinib treatment induces autophagy in MCF-7 and MDA-MB-231 cells, acting as a protective mechanism that enhances cancer cell survival during therapeutic intervention [64]. Interestingly, this protective autophagy process can be further suppressed by lactate, which enhances the effect of gefitinib and promotes cell death [64] however, the authors did not provide a specific mechanism for this observation.

The regulatory influence of lactate on autophagy has led researchers to investigate the impact of lactate-induced lactylation on autophagy processes. Tumor-derived lactate has been shown to elevate H3K18la levels in bevacizumab-treated colon cancer cells, which subsequently upregulates RUBCNL transcription [65]. The RUBCNL protein interacts with BECLIN1 and facilitates autophagosome maturation by mediating the recruitment and function of the class III phosphatidylinositol 3-kinase (PtdIns3K) complex, thereby enhancing autophagy and contributing to cancer progression as well as resistance to bevacizumab therapy [65]. Furthermore, VPS34, a crucial catalytic subunit of the PtdIns3K complex, can also be lactylated at K356 and K781 by the acetyltransferase TIP60, and this lactylation is accompanied by increased autophagic flux and accelerated cancer progression [66]. Interestingly, a recent study revealed a connection between protein lactylation and protein ubiquitination-mediated degradation [62]. Specifically, lactylation of TFEB at K91 inhibits its interaction with WWP2 and prevents TFEB ubiquitination and proteasomal degradation. This modification helps maintain the active form of TFEB, which is essential for elevated autophagy levels in rapidly proliferating cancer cells [62]. In addition, Meng et al. demonstrated that lactate-induced DCBLD1 lactylation, primarily at K172, suppresses its ubiquitination, leading to increased DCBLD1 protein levels [67]. This accumulation of DCBLD1 subsequently inhibits autophagy and impairs the degradation of G6PD via the autophagic lysosomal pathway, ultimately promoting cervical cancer progression [67]. Therefore, the seemingly opposite effects of lactate-driven lactylation on autophagy may largely depend on the specific target proteins being modified. When key autophagy activators are lactylated, the modification enhances their expression or stability, thereby stimulating autophagy; conversely, lactylation of autophagy-inhibitory proteins, such as DCBLD1, enhances their inhibitory function, thereby suppressing autophagy. Nevertheless, despite these opposite effects on autophagy, these types of lactylation events ultimately converge to tumor progression and therapy resistance.

Mitochondrial autophagy, also known as mitophagy, is a form of selective autophagy that facilitates the degradation and removal of damaged or dysfunctional mitochondria through lysosomes and plays a crucial role in preserving mitochondrial and cellular homeostasis [68, 69]. A recent study revealed that mitophagy can also be regulated by lactate-driven lactylation in cancer. In glioma cells, evidence suggests that hypoxia-induced HIF-1α triggers histone lactylation at H3K18la, leading to the upregulation of YTHDF2 expression [70]. The upregulated YTHDF2 then interacts with BNIP3, promoting BNIP3-mediated mitophagy and tumor progression [70].

## Lactate, lactylation and ferroptosis

Ferroptosis, which is characterized by intracellular iron accumulation and lipid peroxidation generation, is a newly identified form of RCD that was first defined in 2012 [71]. Indeed, ongoing research is actively exploring ferroptosis modulation as a novel and promising strategy for anticancer therapy [14, 72]. Moreover, extensive investigations have suggested the intricate interplay between ferroptosis, lactate metabolism, and lactylation, highlighting their collective influence on tumor progression and therapeutic strategies.

Several lines of evidence have presented that lactate, coupled with its metabolites, can confer ferroptosis resistance in tumor cells by modulating multiple pathways within the TME. A previous study showed that lactate supplementation significantly decreased intracellular reactive oxygen species (ROS) levels, reduced lipid accumulation, inhibited the iron level, and upregulated GPX4 expression, which promoted ferroptosis resistance in prostate cancer cells [73]. Moreover, a lactate-induced acidic microenvironment has been shown to drive ferroptosis resistance, a process that can be reversed by sodium bicarbonate, indicating that lactate-mediated ferroptosis resistance is pH dependent [74]. Accordingly, increasing lactate concentrations in the TME can activate the HCAR1/MCT1 pathway, which increases intracellular lactate levels [75]. It has been reported that the increased intracellular lactate within cancer cells can inhibit ACSL4 expression and promote SCD1 expression, which collectively decrease PUFA incorporation while enhancing MUFA synthesis, thereby lowering lipid peroxidation and protecting cancer cells from ferroptosis [75–77]. Additionally, in NSCLC, metabolic reprogramming under therapeutic stress results in drug-induced lactate accumulation, which in turn drives mitochondrial ROS production and activates the P38/SGK1 signaling cascade. This activation reduces the interaction between NEDD4L and GPX4, stabilizing GPX4 levels and enhancing ferroptosis resistance [78]. Consistent with these findings, lactate serves as a metabolic substrate through its conversion to pyruvate, which facilitates NADH accumulation [79]. This NADH is subsequently converted to NADPH through the pentose phosphate pathway, thereby enhancing cellular resistance to ferroptosis by supporting antioxidant defense mechanisms [80]. Taken together, these studies suggest that lactate confers ferroptosis resistance through redox regulation, lipid metabolic reprogramming, pH modulation, and stabilization of key regulators such as GPX4. These mechanisms converge as an adaptive strategy that allows cancer cells to withstand oxidative stress within the hostile TME. Importantly, this highlights lactate metabolism and its downstream signaling pathways as potential therapeutic targets to overcome ferroptosis resistance and enhance anticancer efficacy.

Interestingly, lactate oxidase (LOX catalyzes the oxidation of lactate to pyruvate, generating hydrogen peroxide (H₂O₂) as a byproduct. This H₂O₂-stimulated oxidative stress can significantly promote ferroptosis in tumor cells by amplifying lipid peroxidation through the Fenton reaction [81, 82]. However, lactate exhaustion can further inhibit the HIF-1α/SLC1A1 signaling pathway and suppress GSH production, leading to antioxidant defense and ferroptosis [83]. Therefore, the level of LOX can regulate lactate-related metabolic reprogramming within cancer cells, which in turn influences the sensitivity of tumor cells to ferroptosis.

Beyond its metabolic and signaling roles, lactate also regulates ferroptosis sensitivity by modulating iron homeostasis, a central determinant of this cell death pathway [84]. Cellular iron uptake mainly occurs through transferrin, which transports ferric iron (Fe³⁺) into cells via binding to the transferrin receptor (TFRC) [85]. Notably, lactylation of LSD1 has been shown to enhance its interaction with FosL1, thereby repressing TFRC transcription and suppressing ferroptosis in BRAFi/MEKi-resistant melanoma [86]. In addition to iron transport, iron storage also plays a key role in ferroptosis regulation. Ferritin heavy chain 1 (FTH1), a critical subunit of ferritin, functions as a negative regulator of ferroptosis by sequestering excess iron [87]. In TNBC cell lines, ChIP assays demonstrated that ZFP64 directly binds to the promoters of FTH1 and GCH1, promoting their transcription. Mechanistically, CAFs-derived lactate induces H3K18la modification, which upregulates ZFP64 expression and subsequently suppresses ferroptosis through FTH1-mediated Fe²⁺ depletion and GCH1-mediated inhibition of lipid peroxidation [88]. Besides, lactate-driven H3K18la modification has also been reported to increase NFS1 transcriptional activity in HCC cells under sublethal heat stress [89]. Importantly, NFS1, a key enzyme for Fe–S cluster biosynthesis, has been shown to restrict free iron release and thereby protect cells from ferroptosis [89]. Taken together, these results highlight lactylation as a critical regulator of iron metabolism, enabling cancer cells to evade ferroptosis.

In addition to iron metabolism, recent studies have uncovered a broader role for lactate-derived lactylation in controlling lipid peroxidation and antioxidant defense, two pivotal processes governing ferroptosis sensitivity. In lung cancer, Wu et al. reported the pathological upregulation of glycolysis in cancer cells, an effect that was attributed to elevated H3K18la at the AIM2 promoter region [90]. Specifically, H3K18la-mediated upregulation of AIM2 was further demonstrated to attenuate ferroptosis through the STAT5b/ACSL4 signaling axis [90]. Glutamate-cysteine ligase (GCLC), a key enzyme for glutathione synthesis, suppresses ferroptosis by limiting lipid peroxidation [91]. Recent evidence indicates that lactylation regulates GCLC expression at multiple levels. Specifically, H4K12la has been shown to enhance GCLC transcription in colorectal cancer stem cells [91], while lactate-mediated lactylation of NSUN2 at K508 increases its activity to stabilize GCLC mRNA, thereby conferring ferroptosis resistance in gastric cancer cells [92]. Besides, FSP1 establishes an alternative ferroptosis defense mechanism via reducing CoQ to CoQH2 [93]. Yang et al. demonstrated that inhibition of HDAC decreases HDAC1 lactylation, which reduces m6A modification on FSP1 mRNA. This reduction promotes FSP1 mRNA degradation, thereby lowering FSP1 protein levels and sensitizing cancer cells to ferroptosis [94]. Therefore, lactate-derived lactylation of both histone and non-histone proteins could modify critical ferroptosis regulators, simultaneously controlling lipid peroxidation and antioxidant pathways to suppress ferroptosis and promote tumor cell survival.

Overall, lactate-mediated ferroptosis regulation represents an integrated adaptive network encompassing metabolic, signaling, and epigenetic dimensions. At the metabolic level, lactate serves as both an energy substrate and an antioxidant buffer, fueling NADH/NADPH production and stabilizing redox balance to suppress lipid peroxidation. At the signaling level, lactate upregulates SCD1 and inhibits ACSL4 to remodel lipid metabolism and sustain GPX4 stability, thereby maintaining membrane integrity under oxidative stress. At the epigenetic level, lactate-derived histone and non-histone lactylation coordinate transcriptional programs governing iron metabolism and antioxidant gene expression, reinforcing ferroptosis resistance. However, excessive intracellular lactate accumulation may produce the opposite effect. In particular, inhibition of MCT4, which mediates lactate export, leads to intracellular lactate overload and cytoplasmic acidification. This acidic stress disrupts iron storage homeostasis by promoting FTH1 dissociation and iron release, thereby elevating the labile iron pool and accelerating ferroptosis [95]. In addition, experimental introduction of LOX, which catalyzes the oxidation of lactate to pyruvate, can convert lactate to pyruvate, aggravate hypoxia, and elevate oxidative stress in the TME [96]. This metabolic shift reverses lactate-mediated ferroptosis resistance, enhances lipid peroxidation, and consequently promotes ferroptotic cell death and antitumor immune responses.

Collectively, these findings highlight that both lactate level and its metabolic fate critically determine ferroptosis sensitivity in tumor cells. Therefore, targeting the lactate–lactylation–ferroptosis axis provides a compelling therapeutic opportunity to disrupt this adaptive defense system. By simultaneously intervening in metabolic flux, signaling plasticity, and epigenetic reprogramming, while also considering lactate accumulation and metabolism (e.g., via MCT4 inhibition or LOX-mediated oxidation), such strategies may re-sensitize tumor cells to ferroptosis and overcome resistance to conventional therapies.

## Lactate, lactylation, pyroptosis and cuproptosis

With the development of molecular biology, several newly identified forms of RCD, such as pyroptosis and cuproptosis, have been increasingly studied and may hold significant potential for improving therapeutic efficacy in cancer treatment [15, 16].

Pyroptosis is a lytic and inflammatory type of programmed cell death that is characterized by gasdermin-mediated membrane pore formation, leading to cell swelling, membrane rupture, and the release of pro-inflammatory cytokines such as IL-1β and IL-18 [15]. Recent studies have also shown that the lactate-induced acidic microenvironment stimulates the metabolic enzyme MDH1 to convert α-KG into L-2HG, resulting in elevated intracellular ROS levels in cancer cells [97]. This oxidative stress ultimately triggers pyroptosis via the caspase-8/GSDMC pathway [97]. These findings indicate that lactate may serve as a trigger for pyroptosis, potentially inhibiting tumor progression. Moreover, Zhang et al. identified that gambogic acid treatment recruits the SIRT1, which suppresses CNPY3 lactylation [98]. Consequently, this effect alters the localization and function of CNPY3, leading to lysosomal rupture and the activation of caspase-1/GSDMD-mediated pyroptosis in prostate cancer cells [98]. Overall, the above data indicate the intricate role of lactate metabolism and SIRT1-mediated delactylation in cancer progression, highlighting their selective regulation of pyroptosis in tumor cells.

Cuproptosis, a newly identified copper-dependent form of cell death, is triggered by copper accumulation-induced lipoylated protein aggregation, along with the destabilization of Fe–S cluster proteins, ultimately leading to proteotoxic stress and cell death [99]. Recently, lactate has been identified as a mitochondrial messenger that directly enhances electron transport chain activity independently of its enzymatic conversion by LDH, thereby promoting OXPHOS and concomitantly suppressing glycolysis in a dose-dependent manner [100]. Given that cuproptosis primarily disrupts the mitochondrial respiratory chain, inhibition of glycolysis in tumor cells and the consequent redirection of glycolytic flux toward the TCA cycle can modulate their sensitivity to cuproptosis [101, 102]. This implies that lactate, by promoting a metabolic shift from glycolysis toward mitochondrial respiration, may enhance the vulnerability of tumor cells to cuproptosis [101–103]. Moreover, Zhi et al. constructed a Syr-loaded nanodelivery system (Syr@mPDA@CP), which inhibits lactate efflux and leads to intracellular lactate accumulation within tumor tissues [95]. The resulting lactate accumulation induced intracellular acidification, which in turn suppressed glycolysis [95]. Meanwhile, CP-mediated oxidative stress impeded the compensatory activation of OXPHOS, leading to a profound reduction in cellular ATP production [95]. Notably, the dual inhibition of glycolysis and OXPHOS markedly decreased intracellular ATP levels, which subsequently downregulated the copper transporter ATP7B [95]. These combined effects synergistically enhanced intracellular copper accumulation and ultimately triggered cuproptosis [95].

Building upon these metabolic insights, recent studies indicate that lactate-mediated PTM, especially lactylation, also plays a key role in regulating cuproptosis machinery. FDX1 is the key regulator of cuproptosis, which reduces Cu(II) to Cu(I), thereby promoting protein lipoylation and subsequent dihydrolipoamide S-acetyltransferase (DLAT) aggregation in mitochondria [104]. Recently, Sun et al. presented evidence indicating that METTL16 could promote FDX1 mRNA stability via m6A modification, leading to cuproptosis in gastric cancer [105]. Intriguingly, as a key mediator of cuproptosis, METTL16 undergoes lactylation modification, with lactylation at the K229 site enhancing its methyltransferase activity [105]. Conversely, it was further reported that the SIRT2 protein inhibits METTL16 lactylation, thereby suppressing cuproptosis in gastric cancer [105]. These findings suggest that inducing the METTL16 lactylation could serve as a novel therapeutic strategy for modulating cuproptosis in gastric cancer. Besides, Lin et al. performed RNA-seq and PAS-seq analysis in esophageal squamous cell carcinoma (ESCC) cells, and revealed that lactate treatment resulted in the extension of the 3’ untranslated region (3’UTR) of FDX1 [106]. Further analysis identified that lactate promoted NUDT21 K23 lactylation, leading to 3′ UTR lengthening of FDX1 and ultimately conferred resistance to cuproptosis in ESCC cells [106]. Specifically, lactylation of METTL16 promotes FDX1 expression and sensitizes cells to cuproptosis, whereas lactylation of NUDT21 suppresses FDX1 expression through alternative polyadenylation, thereby conferring cuproptosis resistance. In summary, lactylation serves as a critical regulatory mechanism of cuproptosis, and its functional outcome, whether promoting or inhibiting the process, is determined by the molecular identity of the lactylated substrates. However, given the limited literature on lactylation in the context of cuproptosis, the specific contexts in which lactylation promotes or suppresses cuproptosis require further investigation.

## Effects of RCD on lactate metabolism

Intriguingly, previous studies have investigated the influential role of cell apoptosis in lactate metabolism. For example, Tiefenthaler et al. reported that drug-induced apoptosis increases lactate levels in human leukaemia cells [107]. The authors further noted that this phenomenon results from impaired mitochondrial respiration, leading to a metabolic shift towards glycolysis for lactate production [107]. Conversely, anti-apoptotic genes appear to mitigate this shift by promoting mitochondrial oxidative phosphorylation, thereby limiting lactate accumulation and enhancing its consumption [108, 109]. Therefore, the impairment of mitochondrial oxidative phosphorylation during apoptosis, particularly in the early stages, drives a shift towards glycolysis, resulting in increased lactate levels within tumor cells.

Indeed, oxidative stress initiates autophagy and mitophagy in CAFs, leading to the depletion of functional mitochondria and metabolic reprogramming towards aerobic glycolysis, thereby regulating lactate production and facilitating metabolic crosstalk with tumor cells [110]. For example, Xu et al. revealed that platelet-derived growth factor-BB, which is secreted by tongue squamous carcinoma cells, induces mitophagy in CAFs, leading to the suppression of oxidative phosphorylation, the upregulation of aerobic glycolysis, and elevated lactate secretion [111]. Consistently, several lines of evidence have shown that CAFs overexpressing autophagy-related genes, such as BNIP3, BNIP3L, CTSB, ATG16L1, and MFF, promote the activation of autophagy and mitophagy, ultimately driving a metabolic shift towards aerobic glycolysis and increased lactate production [112–114]. Taken together, these data indicate that targeting autophagy and mitophagy in CAFs represents a promising therapeutic strategy to disrupt tumor-stroma metabolic crosstalk, thereby depriving cancer cells of key metabolic substrates, such as lactate and other bioenergetic fuels.

Nevertheless, the effects of autophagy and mitophagy on glycolytic regulation and lactate metabolism in tumor cells appear to be more complex. A recent study demonstrated that inhibition of autophagy in pancreatic ductal adenocarcinoma cells using 3-MA resulted in elevated intracellular lactate levels [115]. More specifically, another study revealed that impaired Parkin-mediated mitophagy leads to the accumulation of dysfunctional mitochondria in prostate and lung adenocarcinomas. The accumulation of dysfunctional mitochondria results in tumor cells preferentially utilizing aerobic glycolysis for energy metabolism, accompanied by increased lactate production and enhanced lactylation modifications in cancer cells [116]. However, an opposite opinion presented that hepatitis B virus x protein (HBx) induces BNIP3L-mediated mitophagy, which drives a metabolic shift towards glycolysis in HCC cell lines, resulting in increased lactate production and secretion [117]. These findings imply that an imbalance in autophagy or mitophagy, whether through the loss or overactivation of basal autophagy or mitophagy, can disrupt mitochondrial homeostasis in tumor cells, resulting in metabolic reprogramming and alterations in lactate metabolism.

It is well-established that one of the critical metabolic consequences of ferroptosis is the excessive accumulation of ROS, which has been shown to markedly suppress glycolytic activity. Elevated ROS levels can inhibit key glycolytic enzymes such as hexokinase 2 and pyruvate kinase M2, thereby reducing the overall glycolytic flux [118, 119]. This suppression of glycolysis not only compromises ATP production in cancer cells that rely heavily on aerobic glycolysis but also leads to a significant decrease in lactate production [118].

Overall, these data demonstrate that RCD mechanisms dynamically influence lactate metabolism by modulating mitochondrial functionality and glycolytic capacity. Therefore, understanding the interplay between RCD and lactate metabolism may offer novel therapeutic opportunities for the development of combinatorial strategies for cancer treatment.

## Therapeutic implications

In preclinical and clinical studies, the modulation of RCD pathways has emerged as a promising strategy in the development of anticancer therapies. Growing evidence has demonstrated that pharmacologically inducing or inhibiting specific RCD pathways using targeted modulators has significant therapeutic potential, particularly in enhancing the efficacy of anticancer treatments [120]. For example, agents such as venetoclax promote apoptosis by inhibiting the anti-apoptotic protein BCL-2, demonstrating clinical efficacy in treating hematological malignancies [12]. However, tumor cells can develop mechanisms to evade RCD, contributing to therapeutic resistance and tumor progression. As mentioned above, lactate and lactylation modifications are crucial factors in modulating tumor cell resistance to RCD. Therefore, targeting lactate metabolism and lactylation pathways represents a promising strategy to improve the effectiveness of cancer therapies. Specifically, previous publications have documented the clinical impact of lactate and lactylation, highlighting their significant roles in cancer progression and TME remodeling and their potential as therapeutic targets [121–123].

Interestingly, a deeper understanding of the intricate interplay between lactate, lactylation, and the RCD process has driven increasing efforts to develop combination therapies that simultaneously target both metabolic reprogramming and RCD, which holds significant promise for improving the effectiveness of cancer treatments. For example, Chen et al. conducted a platelet biomimetic nanoparticle coloaded with ferroptosis-inducing erastin, superparamagnetic iron oxide nanoparticles and LOX in HCC [77]. In this system, LOX directly converts lactate to pyruvate, thereby depleting intracellular lactate levels and attenuating lactate-mediated resistance to ferroptosis [77]. Simultaneously, the enzymatic reaction generates H₂O₂, which enhances lipid peroxidation through the Fenton reaction, ultimately promoting erastin-induced ferroptosis in HCC cells [77]. Moreover, metformin has been demonstrated to induce RCD in multiple cancer cell lines and suppress cancer progression [124, 125]. However, metformin treatment activates AMPK activity and inhibits mitochondrial complex I activity, leading to impaired OXPHOS and a subsequent lactate production in vitro [123]. Notably, elevated lactate levels, in turn, may impair tumor cell sensitivity to RCD, thereby limiting the overall antitumor efficacy of metformin as a monotherapy [123]. To overcome this limitation, dichloroacetate, a pyruvate dehydrogenase kinase 1 (PDK1) inhibitor, restores mitochondrial respiration by promoting OXPHOS and reduces metformin-induced lactate accumulation in breast cancer cells, thereby enhancing the anti-tumor effect of metformin [123]. Overall, these findings suggest that the interplay between lactate metabolism and RCD pathways has emerged as a novel therapeutic avenue, offering the potential to overcome resistance mechanisms and enhance antitumor efficacy.

Moreover, the accumulation of tumor-derived lactate in the TME also significantly affects the RCD of surrounding immune cells, potentially contributing to immunosuppression. Accordingly, several lines of evidence have shown that tumor-derived lactate in the TME contributes to mitochondrial stress and excessive ROS generation, which promotes the apoptotic death of various immune cells, including cytotoxic T cells and natural killer cells, thereby impairing antitumor immune responses and facilitating immune escape [126, 127]. An additional study revealed that lactate binds to the GPR81 receptor on tumor cells, activating the TAZ protein and enhancing the transcription of PD-L1 [128]. As a result, increased PD-L1 expression on tumor cells interacts with PD-1 receptors on T cells, facilitating T-cell apoptosis and promoting immune evasion [128]. Moreover, Fan et al. reported that sublethal heat treatment enhances lactate secretion in hepatocellular carcinoma cells, which facilitates the M2 polarization of macrophages through reduced paraspeckle formation and inhibits M2 macrophage pyroptosis [129]. As expected, this shift promotes tumor progression by creating an immunosuppressive microenvironment through M2 macrophages [129]. In accordance with this, another study demonstrated that lactate-induced H3K18la increases the transcription level of Parkin by binding to its promoter, thereby promoting Parkin-mediated mitophagy and driving M2 macrophage polarization in bladder cancer, ultimately facilitating immune evasion [130]. Therefore, by interfering with lactate accumulation and its effects on RCD, it may be possible to restore immune cell function, prevent tumor immune escape, and ultimately improve the outcomes of immunotherapy [131].

Nevertheless, despite their potential to provide synergistic therapeutic benefits, these advanced strategies may also raise important clinical considerations. Systemic toxicity remains a major concern. For example, lactate metabolism is critical for physiological functions in the heart, brain, and skeletal muscle, and inhibitors of lactate metabolism, such as LDH or MCT blockers, may disrupt systemic energy homeostasis, causing metabolic disturbances such as fatigue or muscle weakness [23, 132]. Similarly, RCD-targeted agents, such as BCL-2 or GPX4 inhibitors, often lack tumor specificity, which can result in off-target toxicity in normal tissues [133]. Therefore, co-targeting lactate metabolism and RCD pathways may also amplify systemic toxicity. At the same time, tumor heterogeneity adds an additional layer of complexity, as cancer cells display differential reliance on lactate metabolism, and the context-dependent effects of lactate on RCD, either inhibiting or promoting cell death, necessitate precisely timed and context-specific therapeutic interventions. For example, TNBC cell lines display a stronger dependence on glycolysis compared to luminal breast cancer cell lines [134]. Moreover, increased intracellular lactate accumulation has been observed in cisplatin-resistant NSCLC cells compared with parental cells [41], further illustrating that lactate metabolism is dynamically reshaped not only between tumor types but also throughout the course of tumor progression. These observations suggest that lactate-related mechanisms should be explored in a tumor type–specific and stage-dependent manner, which will be crucial for the rational design and clinical implementation of lactate-targeted therapeutic strategies. Moreover, lactate not only shapes cancer cell survival but also modulates the tumor immune microenvironment, suggesting that combined strategies with immunotherapy may be highly effective but require careful optimization to avoid exacerbating immune suppression. To move forward, the lack of validated predictive biomarkers and the complexity of optimal dosing, scheduling, and drug combinations pose significant hurdles for clinical trial design. Although several lactate-targeting therapies, as well as potential agents that modulate novel RCD pathways, have already entered early-phase clinical trials [5, 120], studies specifically evaluating the therapeutic benefit of their combination remain largely absent.

Importantly, these considerations underscore that dynamically regulating lactate metabolism, rather than simply inhibiting it, represents a more nuanced and potentially safer therapeutic approach. In OXPHOS-dependent tumors characterized by high MCT1 expression and functional mitochondria, lactate serves as a critical oxidative substrate that sustains energy production and redox balance. In this context, selective MCT1 inhibition or strategies that restrict lactate availability can effectively deprive tumor cells of metabolic fuel. In contrast, highly glycolytic tumors with elevated MCT4 expression depend on lactate export to prevent intracellular acidification. In these tumors, blocking lactate efflux can induce intracellular acid stress and activate RCD. Nonetheless, such combinations pose the risk of disturbing pH homeostasis in normal tissues, highlighting the importance of spatiotemporal control and selective drug delivery to achieve therapeutic precision.

To address these challenges, increasing attention has been directed toward innovative approaches that may improve therapeutic outcomes, among which responsive nanocarriers represent a particularly promising strategy [96]. Rather than adopting a uniform “one-size-fits-all” suppression, a more adaptive approach that dynamically regulates lactate metabolism in accordance with the fluctuating conditions of the TME may offer greater precision and safety [135]. For example, lactate- or pH-responsive nanoplatforms can synchronize therapeutic activity with tumor-specific metabolic vulnerabilities while sparing normal tissues. A representative design is the FePt@FeOx@TAM-PEG nanoplatform, in which pH-triggered tamoxifen release inhibits mitochondrial complex I in acidic TME, thereby boosting lactate accumulation, intensifying intracellular acidification, and further accelerating nanoparticle disassembly [136]. This positive feedback loop results in a “turn-on” catalytic activity with amplified ROS production selectively within tumors, highlighting the potential of context-dependent regulation to enhance efficacy while minimizing systemic toxicity [136]. In another example, a PEG-CDM surface-modified, GSH-responsive hollow mesoporous organosilica nanoplatform co-delivering hydroxycamptothecin (HCPT) and siMCT-4 cascaded responded to the weakly acidic TME and the elevated intracellular GSH in TME [137]. This sequential responsiveness enabled continuous release of HCPT and siMCT-4, which together induced tumor cell apoptosis and inhibited lactate efflux [137]. The resultant increase in intracellular lactate and depletion of extracellular lactate not only promoted cancer cell death but also reprogrammed the tumor immune microenvironment by shifting tumor-associated macrophages from an M2 to M1 phenotype and restoring CD8 + T cell activity [137]. Collectively, these smart nanoplatforms demonstrate how dynamically regulating lactate metabolism, either by exploiting feedback loops in tumor metabolism or by remodeling the immunosuppressive microenvironment, can provide a powerful framework for integrating lactate-targeted therapies with RCD modulation and immunotherapy. Moreover, benefiting from improved pharmacokinetics and biodistribution, these platforms enable spatially precise regulation of lactate metabolism, site-specific targeting, and controlled drug release, thereby offering new opportunities for synergistic enhancement of anticancer therapies.

## Conclusions, challenges and perspectives

**Fig. 2:**
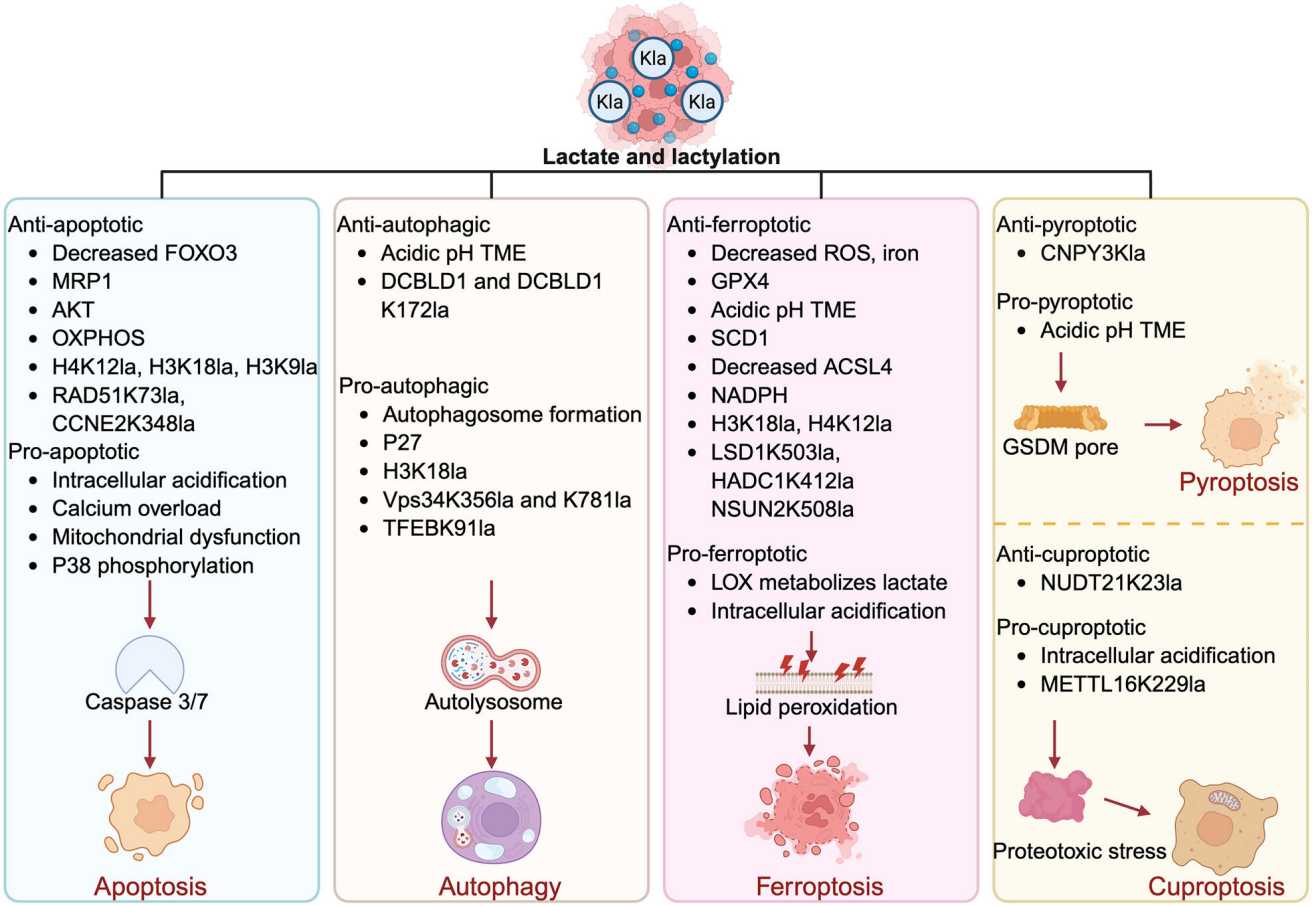
Regulatory roles of lactate and lactylation in different forms of cell death. The dual roles of lactate and lactylation on multiple forms of RCD, including apoptosis, autophagy, ferroptosis, pyroptosis, and cuproptosis. Lactate can regulate cell death by modulating the acidic microenvironment, metabolic reprogramming, and signaling pathways that control critical steps in cell death processes. Moreover, lactate-derived lactylation can also modulate the key gene expression and protein functions involved in cell death pathways. However, their dual roles in regulating cell death are dependent on distinct signaling pathways and metabolic environments within different cell types.

Overall, our review summarizes the critical roles of lactate and lactate-derived lactylation in regulating various forms of RCD programs in cancer cells, including apoptosis, autophagy, ferroptosis, pyroptosis, and cuproptosis (Table 1). As stated above, both lactate and lactylation exhibit dual regulatory roles, capable of either promoting or inhibiting cell death depending on the tumor type, cellular context, and metabolic conditions (Fig. 2).

**Table 1:** Regulatory roles of lactate and lactylation in multiple forms of RCD in tumors.

RCD type	Role of lactate, lactylation	Effect on RCD	Effect on tumor	Tumor type	Cell lines	Year
Apoptosis	Lactate inhibits FOXO3/MAGI1-IT1/miR-664b-3p/IL-6R axis through YTHDF2-mediated m6A modification	Apoptosis ↓	Cisplatin resistance ↑	NSCLC	A549, H1975, and matched cisplatin-resistant cells	2024 [41]
	Lactate upregulates MRP1 via SNAIL/TAZ/AP-1 complex	Apoptosis ↓	Etoposide resistance ↑	NSCLC	A549, H1299	2020 [42]
	1. Lactate activates GPR81/AKT pathway; 2. Lactate provides energy through MCT1	Apoptosis ↓	Erlotinib resistance ↑	NSCLC	PC9, HCC827	2022 [43]
	Lactate activates PI3K/AKT/mTOR/BCL-2 pathway	Apoptosis ↓	Glucose starvation survival ↑	NSCLC, Prostate cancer	A549, H1299, DU145	2015 [44]
	Lactate upregulates BCL-2 and activates AKT	Apoptosis ↓	Glucose starvation survival ↑	Several solid tumors	4T1, Bcap37, RKO, SGC7901	2012 [45]
	Lactate converts glycolysis to OXPHOS	Apoptosis ↓	Glucose starvation survival ↑	Glioblastoma	U251	2018 [46]
	Lactate enhances OXPHOS	Apoptosis ↓	Uprosertib resistance ↑	CRC	HCT116, LS174T	2020 [48]
	Lactate accumulation (resulting from MCT4 inhibition) triggers calcium overload and disrupts mitochondrial function	Apoptosis ↑	Cancer proliferation ↓	NSCLC	H1299, H1975	2023 [49]
	Lactate accumulation (resulting from MCT4 inhibition) lowers intracellular pH and disrupts mitochondrial function	Apoptosis ↑	Cancer proliferation ↓	CRC	CT-26	2022 [50]
	Lactate activates P38 phosphorylation	Apoptosis ↑	Cell death ↑	Cervical cancer	HeLa	2021 [51]
	H4K12la binds to SLFN5 promoter and inhibits its expression	Apoptosis ↓	Cancer proliferation ↑	TNBC	MDA-MB-231, MDA-MB-468	2024 [52]
	H3K18la binds to IDH3G promoter and promotes its expression	Apoptosis ↓	Cancer proliferation, migration, and invasion ↑	NSCLC	H1975	2023 [53]
	H3K18la promotes PPARD/PI3K/AKT pathway	Apoptosis ↓	Cancer proliferation ↑	Breast cancer	MDA-MB-231, MCF-7	2025 [54]
	H3K18la binds to the USP39 promoter, which activates PI3K/AKT/HIF-1α pathway	Apoptosis ↓	Cancer glycolysis, proliferation, migration, and invasion ↑	Endometrial cancer	Ishikawa, KLE	2024 [55]
	H3K9la and RAD51K73la promote homologous recombination repair	Apoptosis ↓	Cisplatin resistance ↑	Ovarian cancer	A2780, SKOV3, OVCAR8, and cisplatin-resistant cells	2025 [57]
	CCNE2K348la inhibits the cell cycle	Apoptosis ↓	Cancer proliferation, migration, and invasion ↑	HCC	Huh7	2023 [58]
Autophagy	1. Lactate promotes autophagosome formation; 2. TFEBK91la inhibits TFEB-WWP2 interaction and TFEB degradation	Autophagy ↑	Cancer proliferation ↑	Several solid tumors	HeLa, PANC	2024 [62]
	Lactate promotes the expression and stability of P27	Autophagy ↑	Glucose starvation survival ↑	Several solid tumors	4T1, Bcap37, RKO, SGC7901	2012 [45]
	Lactate leads to acidic pH TME	Autophagy ↓	Glucose starvation survival ↑	Melanoma	B16F10	2018 [63]
	N/A	Autophagy ↓	Gefitinib resistance ↓	Breast cancer	MCF-7, MDA-MB-231	2018 [64]
	H3K18la binds to the RUBCNL promoter and promotes its expression	Autophagy ↑	Bevacizumab resistance ↑	CRC	HCT116, SW620	2024 [65]
	VPS34 K356la and K781la promote the interaction of Vps34 with Beclin1, ATG14L, and UVRAG	Autophagy ↑	Cancer proliferation ↑	Several solid tumors	N/A	2023 [66]
	1. Lactate activates HIF-1α to enhance DCBLD1 transcription; 2. DCBLD1K172la inhibits DCBLD1 ubiquitination	Autophagy ↓	Cancer proliferation, migration, and invasion ↑	Cervical cancer	C33A, HeLa	2024 [67]
	H3K18la binds to the YTHDF2 promoter and promotes its expression	Mitophagy ↑	Cancer proliferation, migration, and invasion ↑	Glioma	U87	2025 [70]
Ferroptosis	Lactate reduces ROS, lipid accumulation, and iron levels while upregulates GPX4	Ferroptosis ↓	Cancer angiogenesis and proliferation ↑	Prostate cancer	DU145, PC-3	2023 [73]
	Lactate leads to acidic pH TME	Ferroptosis ↓	Cancer survival under hypoxia ↑	Fibrosarcoma	HT1080	2023 [74]
	1. Lactate upregulates SCD1 via AMPK-SREBP1 pathway; 2. Lactate inhibits ACSL4	Ferroptosis ↓	Cancer survival ↑	HCC	Hep3B, Huh-7	2020 [75]
	Lactate upregulates SCD1 via AMPK-SREBP1 pathway	Ferroptosis ↓	Cancer proliferation, migration, and invasion ↑	Esophageal cancer	EC109	2024 [76]
	Lactate upregulates SCD1 and inhibits ACSL4	Ferroptosis ↓	Cancer survival ↑	HCC	H22	2025 [77]
	Lactate inhibits GPX4 ubiquitination via P38/SGK1 pathway	Ferroptosis ↓	Etoposide resistance ↑	NSCLC	A549, H1299	2023 [78]
	Lactate increases NADPH via pentose phosphate pathway	Ferroptosis ↓	Cancer metastasis ↑	Mucosal melanomas	COMM-SUS	2022 [80]
	Lactate is catalyzed by LOX into pyruvate with concomitant H₂O₂ production	Ferroptosis ↑	Cell death, antitumor immunity ↑	Several solid tumors	CT26, MKN45, NUGC4, MFC	2024 [81–83]
	LSD1K503la promotes its interaction with FosL1, which inhibits TFRC transcription	Ferroptosis ↓	Cancer proliferation ↑	Melanoma	A375, and BRAFi /MEKi-resistant A375	2025 [86]
	H3K18la binds to ZFP64 promoter and enhances its expression, thereby upregulating GCH1 and FTH1	Ferroptosis ↓	Doxorubicin resistance ↑	TNBC	BT-549, MDA-MB-231	2025 [88]
	H3K18la binds to NFS1 promoter and enhances its expression	Ferroptosis ↓	Cancer metastasis ↑	HCC	MHCC-97H, Huh7	2025 [89]
	H3K18la binds to AIM2 promoter and enhances its expression, thereby inhibiting STAT5b/ACSL4 axis	Ferroptosis ↓	Cancer proliferation, migration, and invasion ↑	Lung cancer	SK-MES-1, NCI-H1975	2025 [90]
	H4K12a binds to GCLC promoter and enhances its expression	Ferroptosis ↓	Oxaliplatin resistance ↑	CRC	LoVo, SW-620, HCT-116	2025 [91]
	NSUN2K508la stabilizes GCLC mRNA via m5C modification	Ferroptosis ↓	Doxorubicin resistance ↑	Gastric cancer	MKN45	2025 [92]
	HADC1K412la promotes FSP1 mRNA stability	Ferroptosis ↓	Cancer survival ↑	CRC	HCT116, RKO	2025 [94]
	Lactate accumulation (resulting from MCT4 inhibition) lowers intracellular pH, which promotes FTH1 dissociation and iron release	Ferroptosis ↑	Cancer cell death and antitumor immune responses ↑	TNBC	4T1	2025 [95]
Pyroptosis	Lactate leads to acidic pH TME stimulates MDH1 into L-2HG, thereby boosting ROS	Pyroptosis ↑	Cancer proliferation ↓	Several solid tumors	Several cancer cells	2021 [97]
	CNPY3Kla inhibits lysosome rupture	Pyroptosis ↓	Cancer survival ↑	Prostate Cancer	DU145	2024 [98]
Cuproptosis	Lactate accumulation (resulting from MCT4 inhibition) suppresses glycolysis, which inhibits ATP production and downregulating ATP7B expression	Cuproptosis ↑	Cancer cell death and antitumor immune responses ↑	TNBC	4T1	2025 [95]
	METTL16K229la upregulates FDX1 expression via m6A modification on FDX1 mRNA	Cuproptosis ↑	Cancer proliferation ↓	Gastric cancer	HGC-27, AGS	2023 [105]
	NUDT21K23la induces 3’ UTR lengthening of FDX1, thereby inhibiting its protein output	Cuproptosis ↓	Cancer proliferation ↑	Esophageal squamous cell carcinoma	KYSE30	2025 [106]

*NSCLC* non-small cell lung cancer, *CRC* colorectal cancer, *TNBC* triple-negative breast cancer, *HCC* hepatocellular carcinoma, *N/A* information not available.

On the one hand, lactate functions as an alternative energy substrate that fuels the TCA cycle, generates NADH/NADPH, and strengthens antioxidant defenses, thereby inhibiting cell death and sustaining the survival of cancer cells. In addition, lactate can act as a signaling molecule to activate pro-survival signals (such as PI3K/AKT), promote autophagic flux by stimulating autophagosome formation, and enhance anti-ferroptosis ability through multiple pathways, including the stabilization of GPX4 and the inhibition of ACSL4 expression. Moreover, histone and non-histone lactylation epigenetically reprogram cell death gene expression (e.g., VPS34, FTH1, GCLC), further supporting tumor adaptation to stress. On the other hand, intracellular lactate accumulation can also lead to acidosis and oxidative stress, impairing mitochondrial function and triggering cell death.

The apparent bidirectional effects of lactate largely depend on its source and metabolic context, including whether it is self-produced by tumor cells or accumulated due to altered transport or metabolic consumption. Under stress conditions such as hypoxia or drug exposure, tumor cells often upregulate glycolysis and self-produce lactate as a compensatory mechanism to resist death. In this setting, lactate efflux via MCT4 maintains intracellular pH homeostasis and prevents acid-induced cytotoxicity. However, when MCT4 is experimentally inhibited, tumor cells lose this buffering capacity, resulting in excessive lactate accumulation and intracellular acidification. This in turn collapses the mitochondrial membrane potential, activates caspase-dependent apoptosis [49, 50], and increases the labile iron pool, thereby promoting lipid peroxidation and ferroptosis [138]. Thus, the manipulation of lactate flux can completely reverse the effect of lactate from cytoprotective to cytotoxic. Such findings are consistent with recent nanotherapeutic studies where blocking lactate export induced metabolic stress and sensitized tumor cells to cell death [137]. In addition, manipulating lactate metabolism through the introduction of LOX, which converts intracellular lactate to pyruvate while producing H₂O₂, artificially enhances oxidative stress and sensitizes tumor cells to ferroptosis. Given that many tumors exhibit high lactate concentrations (10 to 40 mM) in the TME, this metabolic feature can be exploited by LOX-based strategies to selectively induce ferroptosis in cancer cells [139].

Furthermore, tumor heterogeneity, particularly in mitochondrial oxidative capacity, determines the outcome of lactate-mediated regulation of cell death. Tumor cells with intact OXPHOS machinery and high MCT1 expression can efficiently oxidize lactate to pyruvate, fueling the TCA cycle and generating NADH/NADPH to maintain ATP and antioxidant defenses, thereby suppressing cell death. In contrast, highly glycolytic tumors with defective OXPHOS or low MCT1 expression exhibit impaired capacity to metabolize lactate, and its accumulation under these conditions tends to cause intracellular acidification, Ca²⁺ overload, and mitochondrial collapse, ultimately favoring lactate-induced cell death. For example, in Chinese oral mucosal melanoma (COMM), cells display heterogeneous metabolic phenotypes: the adhesive subtype (COMM-AD) exhibits high MCT4 expression and relies predominantly on glycolysis, whereas the suspension subtype (COMM-SUS) expresses high levels of MCT1, enabling efficient lactate uptake that enhances NADPH generation, thereby inhibiting ferroptosis and promoting cancer metastatic potential [80].

In addition, the dichotomous role of lactate also emerges during tumor progression, reflecting the dynamic metabolic reprogramming capacity of cancer cells. In parental A549 cells that lack lactate-dependent protective mechanisms, excessive lactate accumulation triggers ER stress and induces apoptosis [140]. In contrast, cisplatin-resistant A549 cells, which have undergone long-term selective pressure and exhibit elevated intracellular lactate, have established a lactate-dependent survival program in which lactate signaling suppresses apoptosis and enhances chemoresistance [41]. A similar bidirectional regulation is observed in autophagy under glucose deprivation. Within the first 48 h of glucose deprivation, lactate suppresses autophagy, whereas prolonged exposure beyond 48 h leads to autophagy activation, reflecting a late-phase metabolic adaptation [45]. These observations highlight that metabolic reprogramming allows tumor cells to modulate the effect of lactate on cell death, determining whether it functions as a survival-promoting or cytotoxic signal.

Moreover, the genetic background of tumor cells (e.g., p53, PTEN) markedly influences how lactate or lactylation modulates death pathways, explaining why the same metabolic perturbation can yield opposite outcomes across different tumor models. Last but not least, when lactate accumulation exceeds the buffering capacity of the extracellular matrix, local acidosis occurs, causing oxidative stress and mitochondrial dysfunction. In this situation, lactate shifts from a metabolic fuel to a toxic byproduct, ultimately leading to cell death. This explains why experimental treatment with high concentrations of lactate, such as 80 mM, can trigger rapid cell death [44].

**Fig. 3:**
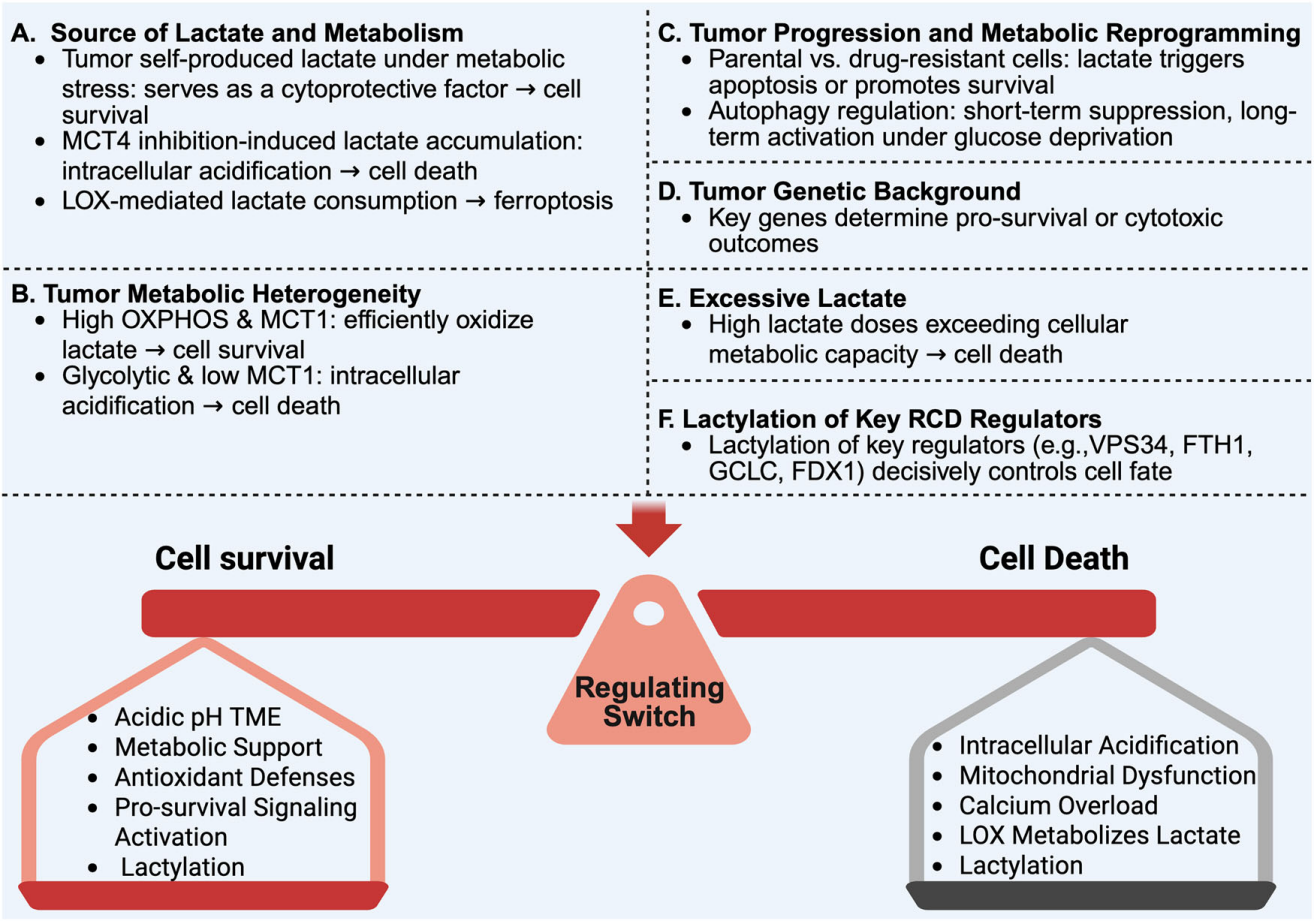
Bidirectional regulation of tumor cell fate by lactate and lactylation. Lactate and Lactylation exert context-dependent effects on tumor cells, functioning either as a pro-survival signal or as a cytotoxic signal. The dual effects of lactate and lactylation are determined by multiple factors.

Beyond its metabolic effects, lactate also regulates cell fate through protein lactylation, including both histone and non-histone modifications. For example, lactylation of METTL16 promotes FDX1 expression and sensitizes cells to cuproptosis [105], whereas lactylation of NUDT21 suppresses FDX1 expression, thereby conferring cuproptosis resistance [106]. These findings highlight that lactylation of key cell death regulators can serve as a decisive determinant of cell fate. The complex and context-dependent effects of lactate and lactylation on tumor cell survival and death are summarized in Fig. 3.

As a newly discovered modification, the existing research on lactylation is still at an initial stage. Nowadays, with the rapid advancement of MS/MS-based proteomic technologies, comprehensive analyses have systematically summarized Kla sites and responsive proteins across multiple species [141], highlighting the evolutionary conservation and functional versatility of lactylation as a PTM. A recent study generated the first comprehensive lactylation landscape of HCC by performing a proteomic survey of Kla modifications in normal liver tissues, non-metastatic HCC, and HCC with lung metastasis [142]. In total, 2045 Kla-modified sites derived from 960 proteins were identified, providing valuable insights into the global regulatory network of lactylation in HCC progression [142]. Similar lactyl-proteomic analyses have been successively reported in other malignancies, such as TNBC and colorectal cancers [143, 144]. These findings underscore the potential of protein lactylation as a biomarker as well as a target for the development of innovative cancer therapies. Moreover, emerging single-cell sequencing and spatial omics technologies offer the opportunity to map lactylation at cellular resolution, enabling the investigation of its spatial distribution, heterogeneity, and context-specific functional roles within the TME [145, 146]. Based on these advances, we believe that integrating spatially resolved lactyl-proteomics with functional analyses will provide critical insights into tumor metabolism and cell fate regulation, ultimately guiding the development of more precise cancer therapies.

Overall, the emerging insights into the regulatory roles of lactate and lactylation modifications in tumor cell death provide a novel and promising framework for understanding tumor progression and therapeutic resistance. Accumulating evidence suggests that both lactate and lactylation exert context-dependent, dual effects on multiple forms of RCD. This review not only broadens our knowledge of the crosstalk between metabolic reprogramming and cell death pathways, but also underscores the potential of targeting the lactate–lactylation–cell death axis as a therapeutic strategy for cancer treatment in the future.
